# Go with the flux: Modeling accurately predicts phenotypes of *Arabidopsis* lipid mutants

**DOI:** 10.1093/plphys/kiae620

**Published:** 2024-11-22

**Authors:** Erin Cullen, Maneesh Lingwan

**Affiliations:** Assistant Features Editor, Plant Physiology, American Society of Plant Biologists; Assistant Features Editor, Plant Physiology, American Society of Plant Biologists; Donald Danforth Plant Science Center, St. Louis, MO 63132, USA

Mutations serve as a foundation for change in genetic material and are crucial forces driving evolution, enabling adaptation to diverse environments. Plant mutagenesis has been utilized as an essential tool kit for studying the genetics behind various biological traits. The phenotypic analysis of these mutants provides insights into how specific genes influence biochemical and physiological characteristics. *Agrobacterium*-mediated T-DNA insertion mutagenesis is one of the prominent techniques in *Arabidopsis thaliana*, and it has been instrumental in characterizing growth, transcript, and metabolic phenotypes (O’Malley et al. 2015). Despite these advances, challenges remain to associate specific visible and biochemical phenotypes with mutated genes.

Lipids represent a class of structurally diverse molecules essential for building membranes, energy reserves, and safeguarding plants under environmental stress. Arabidopsis mutant collections and their associated databases are invaluable in distinguishing the genes involved in lipid metabolism and their metabolic networks (McGlew et al. 2015). In a metabolic network, metabolites play an important role in biochemical reactions and can be seen as nodes, while the transformation of one metabolite to another is represented by edges, which indicate the metabolic flux. Previously, the metabolite flux-sum analysis approach demonstrated the use of a quantitative measure of metabolite known as the flux-sum to indicate the turnover rate of a particular metabolite and demonstrated key variables in metabolic models (Chung and Lee 2009). Lipid metabolic networks explaining metabolic phenotypes have been obtained through reductionist approaches in the past. However, with advancements in technology, high-throughput computational tools and mathematical frameworks can be utilized to predict accurate phenotypes. Plant Lipid Module (PLM) is an automated tool that operates within a constraint-based modeling framework that can predict a mechanistic description of lipid metabolism in plants (Córdoba et al. 2023).

In this issue of Plant Physiology, Córdoba et al. (2024) used a data-integrative modeling approach to predict the biochemical and physiological consequences of mutations in *A. thaliana* lipid metabolism. The authors applied their PLM to 64 Arabidopsis T-DNA lines for the mechanistic reconstruction of the lipid metabolic network in the *A. thaliana* rosette leaf. They tested how accurately the PLM can identify mutated genes and predict the phenotypic effects of mutations at the key steps of the lipid biosynthesis pathway. The authors validated predictions with relative abundances of lipid profiles measured in this study and in previously published results (Lusk et al. 2022). Integrating lipid abundances into the PLM demonstrated that the constraint-based model successfully predicted changes in the rosette growth phenotype of 73% of mutants. Although 27% of the predictions did not match with published phenotypes, the lack of accuracy can be linked to several other metabolic intermediates that contribute to growth and lipid biomass. For biochemical phenotypes, the PLM accurately anticipated alterations of fluxes in 49% of the T-DNA lines; inconsistencies between measured and reported lipid profiles, as well as inadequate experimental data for some lipids might contribute to the low predictive ability of the PLM for biochemical phenotype. Importantly, the model is capable of identifying small differences in lipid abundance, which makes it possible to characterize weak phenotypes (Fig. 1).

**Figure 1.**
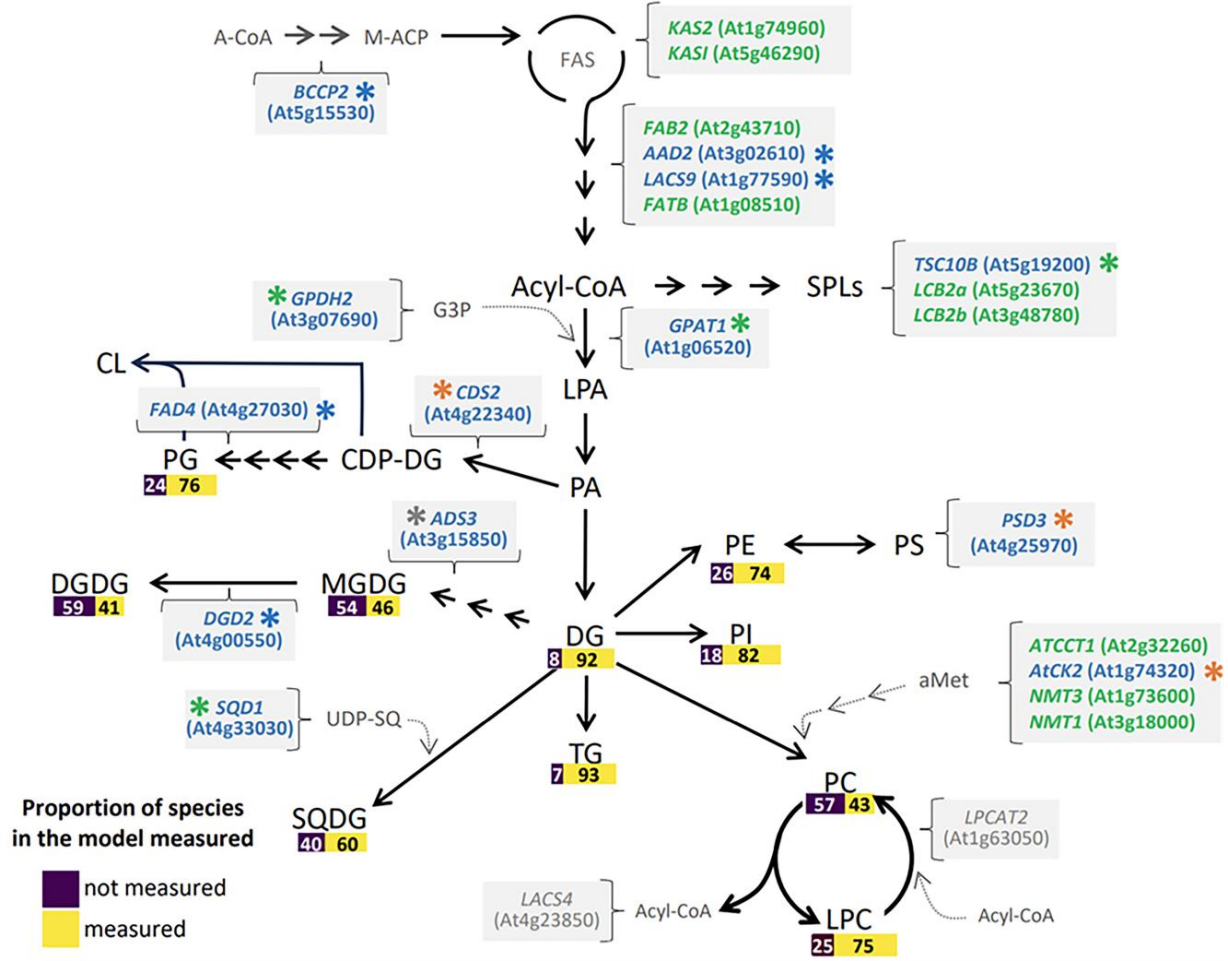
The lipid metabolic networks affected by selective Arabidopsis T-DNA mutations are illustrated. Each metabolic step represents a group of mutants that were characterized biochemically in-house by measuring lipid profiles. The purple and yellow boxes in the pathway show the percentages of the measured species in relation to the total species for each lipid class included in the PLM. Different font colors indicate the mutant loci that the PLM simulation predicted to see changes in biochemical phenotype. Green indicates predictions consistent with phenotypes reported in the literature, while blue represents predictions that disagree with the literature reports. Gray indicates mutants for which it was not possible to find phenotypic information. Asterisks denote the phenotype after adding lipid class constraints to the model. Green asterisks identify mutants for which incorporating constraints accurately reflect the behavior of the simulated phenotype. Orange asterisks signify cases where adding constraints were a small help to match the reported phenotype. Blue asterisks indicate that constraints in the lipid class did not improve previously achieved results. The figure was adapted from Córdoba et al. (2024). Abbreviations: A-CoA, acetyl CoA; SPLs, sphingolipids; CDP-DG, CDP diacylglycerol; CL, cardiolipin; DG, diacylglycerol; DGDG, digalactosyldiacylglycerol; FAS, fatty acid synthase complex; LPA, lysophosphatidic acid; MGDG, monogalactosyldiacylglycerol; PA, phosphatidic acid; PC, phosphatidylcholine; PE, phosphatidylethanolamine; PG, phosphatidylglycerol; PI, phosphatidylinositol; PS, phosphatidylserine; SQDG, sulfoquinovosyl diacylglycerol; CHOs, carbohydrates; and FC, fold change.

A challenge in understanding plant lipid metabolism is that lipid mutants often show no phenotype due to gene redundancy, tissue, or development-specific lipid composition. A literature review by the authors found that 84% of the T-DNA mutants examined in this study did not show a growth phenotype and that 44% did not display a biochemical phenotype. The authors hypothesized that this is due to compensation through additional metabolic pathways. To investigate this hypothesis, they interrogated published gene expression data for the *glycerol-3-phosphate sn n-acyltransferase 1* (*gpat1*) mutant. They found upregulation of genes involved in the biosynthesis of polysaccharides, wax, cutin, and secondary metabolites such as isoprenoids. Simulations supported the above data (i.e. matched the direction of the flux), indicating that compensation may be taking place.

This study exhibits the power of taking a systems biology approach to predict the growth and lipidome phenotype of lipid metabolism mutants. New knowledge regarding the lipid metabolism pathway and experimental data can be used to refine the model and allow predictions with enhanced accuracy in the future. Given that lipid composition varies between different plant organs, it would be interesting to apply this approach to additional organs, such as flowers and seeds. Finally, this strategy could be particularly valuable when expanded to agronomically important species to generate a list of candidate genes for experimental validation.

## Data Availability

No data were generated or analyzed in this study.
